# Myofascial force transmission between the ankle and the dorsal knee: A study protocol

**DOI:** 10.1371/journal.pone.0276240

**Published:** 2022-11-03

**Authors:** Lisa Mohr, Lutz Vogt, Michael Behringer, Jan Wilke

**Affiliations:** 1 Department of Sports Medicine and Exercise Physiology, Institute of Sports Sciences, Goethe University Frankfurt, Frankfurt/Main, Germany; 2 Division of Health and Performance, Institute of Occupational, Social and Environmental Medicine, Goethe University, Frankfurt/Main, Germany; 3 Department of Movement Sciences, University of Klagenfurt, Klagenfurt am Wörthersee, Austria; Ningbo University, CHINA

## Abstract

**Background:**

Connective tissue links the skeletal muscles, creating a body-wide network of continuity. A recent in-vivo experiment demonstrated that passive elongation of the calf caused a caudal displacement of the semimembranosus muscle, indicating force transmission across the dorsal knee joint. However, it remains unclear as to whether this observation is dependent on the joint angle. If force would not be transmitted at flexed knees, this would reduce the number of postures and movements where force transmission is of relevance. Our trial, therefore, aims to investigate the influence of passive calf stretching with the knee in extended and flexed position on dorsal thigh soft tissue displacement.

**Methods:**

Participants are positioned prone on an isokinetic dynamometer. The device performs three repetitions of moving the ankle passively (5°/s) between plantar flexion and maximum dorsiflexion. With a washout-period of 24 hours, this procedure is performed twice in randomised order, once with the knee extended (0°) and once with the knee flexed (60°). Two high-resolution ultrasound devices will be used to visualize the soft tissue of the calf and dorsal thigh during the manoeuvre. Maximal horizontal displacement of the soft tissue [mm] during ankle movement will be quantified as a surrogate of force transmission, using a frame-by-frame cross-correlation analysis of the obtained US videos.

**Discussion:**

Understanding myofascial force transmission under in-vivo conditions is a pre-requisite for the development of exercise interventions specifically targeting the fascial connective tissue. Our study may thus provide health and fitness professional with the anatomical and functional basis for program design.

**Trial registration:**

The study is registered at the German Clinical Trials Register (TRN: DRKS00024420), registered 8 Februar 2021, https://www.drks.de/drks_web/navigate.do?navigationId=trial.HTML&TRIAL_ID=DRKS00024420.

## Introduction

It is a long-held belief that fascia, the collagenous connective tissue surrounding the skeletal muscle, represents a passive packing organ with limited significance for the locomotor system [1]. However, recent research has not only revealed its contribution to proprioception and pain [2–4]. Beyond providing sensory input [2–4], fascial tissue has been demonstrated to connect both, parallelly [5] (e.g. gastrocnemius and soleus) and serially [6, 7] (e.g. gastrocnemius and Hamstrings) arranged muscles. Following this paradigm, Wilke et al. [6] found strong evidence for the existence of a posterior myofascial chain, consisting of the plantar fascia, Achilles tendon, gastrocnemius muscle and fascia, Hamstring muscles and fasciae, sacrotuberous ligament, lumbar fascia, and erector spinae muscle and fascia. Interestingly, biomechanical experiments in cadavers showed that significant forces may be transmitted via the described tissue continuities [8, 9]. Yet, due to fixation of cadavers using solutions such as formaldehyde, the findings cannot be readily interpolated to in-vivo conditions.

Only few studies have investigated myofascial chains and their mechanical relevance in the living organism. Most of these found non-local improvements of range of motion (ROM) after stretching or self-massage applied at distant, structurally connected body locations [10–12]. However, as ROM represents a functional outcome, these observations cannot be considered as valid proofs of force transmission. With a pioneering experiment, Crus-Montecinos et al. [13] provided initial direct evidence for a mechanical interaction of serially connected muscles, revealing by means of high-resolution ultrasound imaging that an anterior pelvic tilt is associated with an cranial displacement of the deep fascia of the gastrocnemius. Hereafter, Wilke et al. [14] first investigated myofascial force transmission in the opposite (caudal to cranial) direction. In an exploratory trial, they examined the impact of ankle motion on soft tissue displacement of the dorsal thigh. In a prone position and with the knee extended, the ankle of eleven healthy individuals was passively moved between plantar flexion and dorsal extension by an isokinetic dynamometer. Simultaneous ultrasound recordings revealed a caudal displacement of the semimembranosus muscle. Despite these intriguing findings, the trial of Wilke et al. [15] had a small sample size and no control condition.

Movements like walking, squatting or jumping require a change and interaction between flexion and extension, especially in the ankle and knee joints. From a theoretical point of view, to transmit force from the calf muscles to the posterior thigh, the tissue connection between the gastrocnemius and the Hamstrings needs to be stiffened. Anatomically, the gastrocnemius attaches to the medial and lateral epicondyles of the femur. In view of the resulting biarticularity, extension of the knee leads to a higher stretch of the gastrocnemius when compared to flexion of the knee [16]. As the gastrocnemius and its fascial connection to the Hamstrings are lengthened and stiffened when approaching knee extension while becoming slack when approaching knee flexion, we expect a higher degree of force transmission at knee extension. So far, it is unknown as to whether myofascial force transmission is dependent on the joint position. Knowledge about this factor is, however, crucial: if no forces would be transmitted at flexed knees, this would mean that the number of postures, movements and situations where force transmission is of relevance, is reduced. As a consequence, the planned study aims to investigate the influence of passive calf stretching with the gastrocnemius in fully elongated (knee extended) and slack (knee flexed) position on dorsal thigh soft tissue displacement.

We hypothesize that (1) passive ankle motion at knee extension causes caudal displacements of the Hamstring muscles while little or no displacement can be seen at knee flexion and (2) the degree of Hamstring muscle displacement is dependent on ankle movement.

## Materials and methods

### Study design

**Fig 1:**
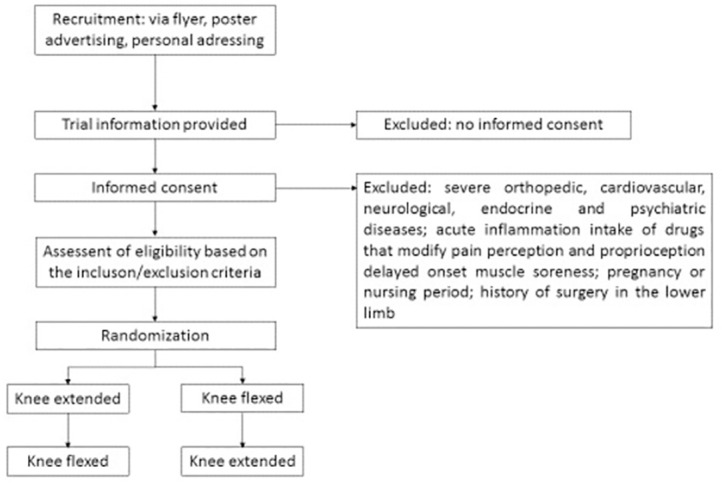
Flow of participants.

The trial adopts a randomized crossover design (Fig 1). All enrolled participants will undergo two examinations, which are separated by a washout period of 24 hours: Displacement of the semimembranosus and its fascia upon ankle joint motion will be examined with the knee in (1) extended and (2) flexed position. To exclude possible influences of circadian rhythm, the examinations will be performed at the same daytime and in the same room.

### Sample

Healthy active individuals, both males and females, aged between 18 and 40 years will be enrolled. Exclusion criteria are defined as severe orthopedic, cardiovascular, neurological, endocrine and psychiatric diseases, acute inflammation, intake of drugs that modify pain perception and proprioception, delayed onset muscle soreness, pregnancy or nursing period and history of surgery in the lower limb. Flyer, poster advertising, as well as personal addressing will be used for recruitment.

### Ethics approval and consent to participate

The trial will be conducted according to the Guidelines of Good Clinical Practice and according to the Declaration of Helsinki including its recent modification of Fortaleza. The local ethics committee of Goethe University Frankfurt (2020-71G) has obtained ethical approval on 23^rd^ December 2020. All participants will provide written informed consent. The collection, transfer, storage and analysis of personal data will be performed in accordance with applicable law.

### Patient and public involvement

Patients and Public were not involved in the design and conduct of the study, as well as in the choice of outcome measures and recruitment.

### Experimental approach

**Fig 2:**
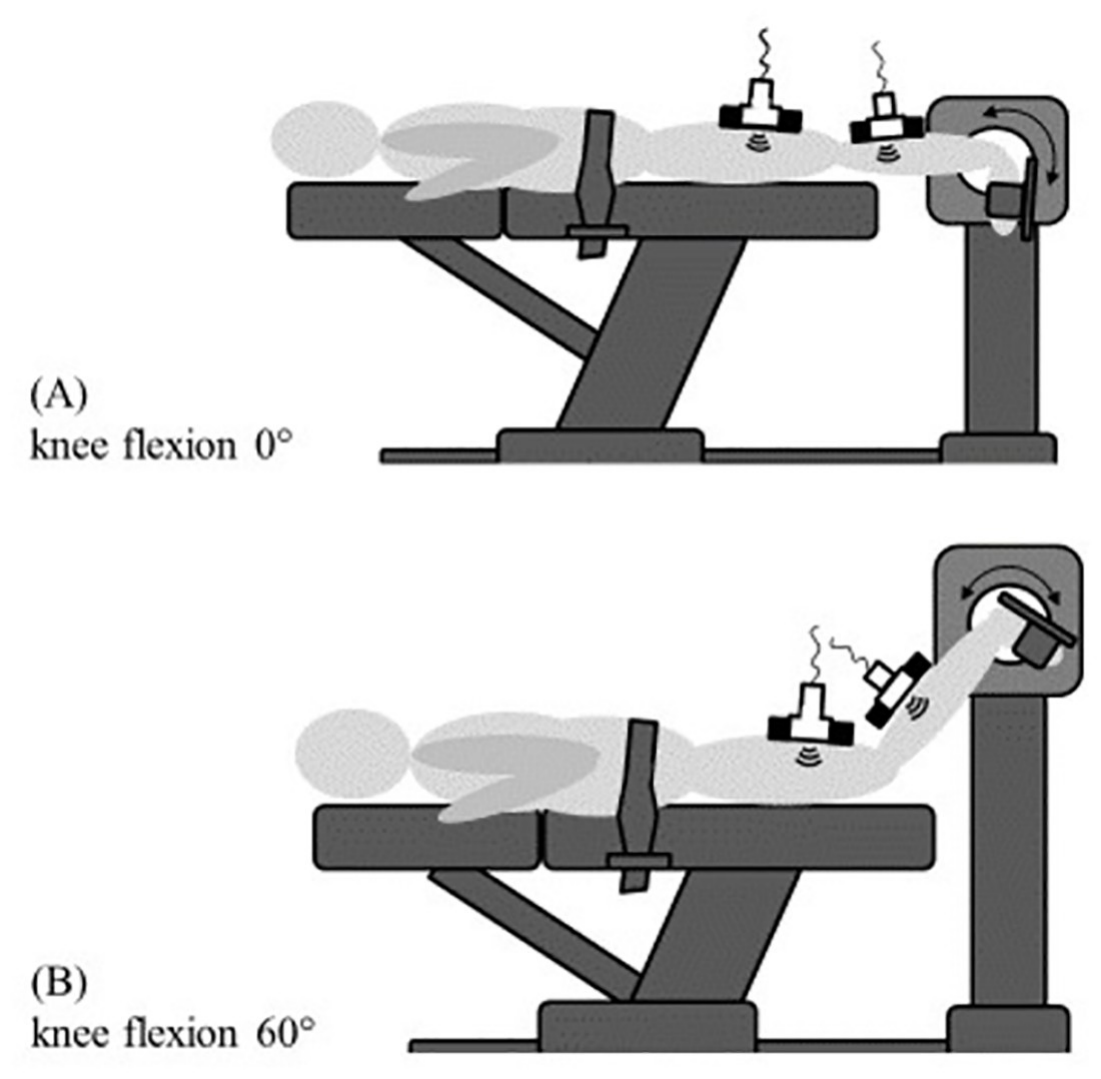
Experimental approach. All participants will undergo two testing conditions. In randomized order, in prone position, the ankle will be moved between plantar flexion and dorsal extension by use of a dynamometer (arrows) with the knee in extended (A) and flexed (B) position. In both conditions, ultrasound recordings of the calf and dorsal thigh will be made to estimate tissue displacement induced by the ankle motion.

Participants are positioned prone on an isokinetic dynamometer (IsoMed 2000, D. & R. Fersti GmbH, Hemau, Germany). The ankle (tested leg chosen randomly) will be moved passively from plantar flexion to the maximal achievable dorsal extension using the continuous passive motion function, which constantly moves the joint through the set range of motion. Based on previous studies, after a warm-up of three plantar flexion-extension cycles [8], three repetitions are performed at an angular velocity of 5°/s [13, 15]. Secondary movement of the knee and the pelvis is minimized by means of velcro straps. The participants are instructed to avoid any voluntary muscle activity. To verify muscle inactivity, surface electromyography (sEMG) (BioNomadix EMG2, Biopac Systems Inc., Goleta, CA, United States) of the semimembranosus and gastrocnemius medialis will be applied. Additionally, sEMG will be used to provide the participants with live biofeedback. To reduce impedance between electrodes and the skin, the area of application will be shaved and cleaned. According to the SENIAM guidelines [17], electrodes will be placed on the most prominent bulge of the gastrocnemius medialis and semimembranosus muscles. In order to identify the correct locations for electrode placement, ultrasound (US) will be used. All electrodes will be placed with an inter-electrode distance of 2 cm and following the longitudinal axis of the muscle [17]. All included individuals, in randomized order, undergo the above described assessment procedure twice. In one session, the knee is fixed in extension (0° knee angle) while in the other condition, the knee is fixed in 60° flexion. This angle was chosen, because the gastrocnemius has been shown to be slack in this position [18]. A schematic depiction of the experimental approach is shown in Fig 2.

To visualize tissue displacement upon ankle movement, two high-resolution ultrasound devices (Siemens Acuson X300 PREMIUM EDITION, Siemens Medical Solutions USA, Inc., Mountain View, CA and Siemens Acuson Redwood, Siemens Healthcare GmbH, Erlangen, Germany) will be used. Video recordings are made using linear array transducers (VF10-5 linear array, 5,0–10,0 MHz, Siemens Medical Solutions USA, Inc., Mountain View, CA and 10L4 linear array, 3,3–11,4 MHz, Siemens Healthcare GmbH, Erlangen, Germany) positioned over (1) the gastrocnemius and (2) the semimembranosus muscle.

### Data processing and outcome

**Fig 3:**
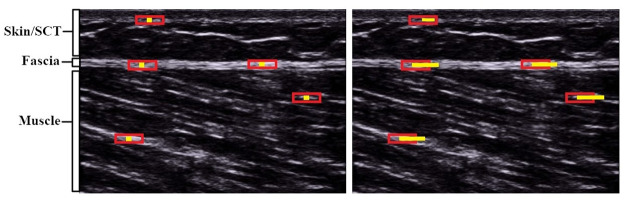
Exemplary visualization of US cross-correlation analysis. Here, five ROIs (red rectangles) have been positioned at rest (left image): three within the fascia and one in each, the subcutaneous tissue (SCT) and the muscle ROIs. The small yellow squares within the ROIs represent the center of the non-moving ROIs. Upon movement (right image), pixel displacement relative to the center of the non-moving ROIs are tracked (yellow line in right picture). The end of the line indicates the maximal displacement, computed by the software algorithm.

The maximal horizontal displacement [mm] of the semimembranosus will be quantified using cross-correlation analysis of the videos visualizing the dorsal thigh. Briefly, within pre-defined regions of interest (ROI), the used MATLAB (The MathWorks, Inc., Natick, MA, United States) based algorithm [19] determines the correlation coefficient between the pixel gray levels of successive frames (Fig 3). The algorithm of Dilley et al. [19] has been shown to represent a reliable method to quantify tissue displacement (ICC .7 to .99). High precision (error of 0.1 to 0.3 mm for displacements between 1 and 3 mm) of the algorithm has been demonstrated [19]. In the planned trial, six equidistant ROIs will be used. Excellent reliability of this approach has been demonstrated before [8, 15].

Besides measuring semimembranosus displacement, we will examine the displacement [mm] of the gastrocnemius muscle during the ankle movement. Using the same cross-correlation analysis as described before. In the videos of the calf, four ROIs will be placed in the lower aponeurosis of the gastrocnemius muscle.

The recorded US data and the ROM measurements of the dynamometer, as well as the EMG data will be synchronized using a common interface (AcqKnowledge, Biopac Systems Inc., Goleta, CA, United States). To detect unwanted muscle activity, the implemented function of the interface to locate muscle activity will be used. First, the algorithm determines the mean and standard deviation of the EMG signal under resting conditions over a 0.25 s period. Based on the algorithm of Hodges and Bui [20], a filtered average rectified value (AVR) will be computed. The variance with regard to the noise will be extracted dividing the difference between the ARV and the mean by the standard deviation. For the resulting signal, the median is calculated for the entire waveform and any activity exceeding this median for at least 0.1 s will be considered as muscle activity [20] and will be excluded from the analysis.

## Statistics

With regard to the dynamometer (ankle angle in °) and ultrasound data (tissue displacement in mm), mean values of the three repetitions are calculated for both conditions (knee extended/flexed) and muscles. To answer our first hypothesis (higher tissue displacement of the dorsal thigh in the condition with the knees extended), a paired t test for dependent samples (or a Wilcoxon test in case of non-normal data distribution) will be computed in order to reveal systematic differences between the two test conditions. To estimate the clinical relevance of the findings, effect sizes will be calculated and interpreted according to Cohen [21] as small (d = .2), medium (.5) or large (.8). Additionally, for hypothesis two, the possible association between ankle movement/ gastrocnemius displacement and dorsal thigh tissue displacement will be examined using linear regression (dependent variable: tissue displacement). Normal distribution will be checked by using the Kolmogorov-Smirnov-Test for the regression residuals, in case of non-normal distribution data, bootstrapping will be applied. All calculations will be made with SPSS 20 (IBM, Armonk, USA); the significance level will be set to α = .05.

According to Faul et al. [22], an a priori biometric sample size calculation was performed, using the algorithm of G*Power, version 3.1.5 (Heinrich-Heine University of Düsseldorf, Germany) for each hypothesis. The computation showed, hypothesis one to require n = 29 individuals (α = .05, 1-ß = .8, Cohen’s d = .5, Dropout: 5%) and hypothesis two to require n = 56 participants (α = .05, 1-ß = .8, Cohen’s f² = .15, Dropout: 5%). Consequently, the enrollment of n = 56 individuals will ensure sufficient power to detect both, significant differences (hypothesis 1) and associations (hypothesis 2) with medium effect sizes according to Cohen [21].

## Discussion

The relevance of the extramuscular connective tissue for locomotor mechanics represents one of the main focuses of fascia research [23]. However, evidence on force transmission, hitherto, has mostly been derived from cadaver studies and experiments using functional outcomes (i.e. ROM, [8, 10–12]). As a consequence, it is unclear if observed remote exercise effects (e.g. flexibility increases) are due to mechanical interactions or neural adaptations such as a systemic reduction of stretch tolerance [24]. Against this background, the planned trial will help to more conclusively delineate the contribution of tissue continuity. Its main purpose is to investigate the influence of passive calf stretching with the knee extended or flexed on dorsal thigh soft tissue displacement.

The results of the planned trial, will also provide more definitive practical insights into how stretching transmits force to adjacent structures by visualizing remote tissue behavior as a function of joint angle. With regard to the dorsal knee, it will particularly allow the identification of movements and situations where myofascial force transmission may play a role or not. From a more general point of view, therapists and exercise professionals count among the groups that may benefit from our findings. Limitations in ROM have been associated with several musculoskeletal pathologies such as groin pain [25] or low back pain [26]. Traditionally, related active treatments have been based on the application of local flexibility treatments. However, as a couple of studies have demonstrated the existence of non-local exercise effects following clinical treatments based on myofascial chain concepts [10–13], our study may provide further arguments for their use.

Our study has several methodological strengths. For instance, first, it is based on very similar pilot works and a robust sample size calculation, allowing sufficient power to detect clinically relevant force transmission effects. Second, it uses established procedures and valid devices (e.g. isokinetic dynamometer) to control for potentially relevant confounders such as involuntary muscle activation (EMG measurements) or probe displacement (ultrasound-based movement analysis system).
